# Optimal dose of fenfluramine in adjuvant treatment of drug-resistant epilepsy: evidence from randomized controlled trials

**DOI:** 10.3389/fneur.2024.1371704

**Published:** 2024-03-25

**Authors:** Yingchun Xu, Deng Chen, Ling Liu

**Affiliations:** Department of Neurology, West China Hospital, Sichuan University, Chengdu, Sichuan, China

**Keywords:** fenfluramine, dose-related, drug-resistant epilepsy, DRAVET syndrome, Lennox–Gastaut syndrome

## Abstract

**Objective:**

Several clinical trials have suggested that fenfluramine (FFA) is effective for the treatment of epilepsy in Dravet syndrome (DS) and Lennox–Gastaut syndrome (LGS). However, the exploration of its optimal target dose is ongoing. This study aimed to summarize the best evidence to inform this clinical issue.

**Materials and methods:**

We searched PubMed, Embase (via Ovid), and Web of Science for relevant literature published before December 1st, 2023. Randomized, double-blind, placebo-controlled studies that evaluated the efficacy, safety, and tolerability of FFA in DS and LGS were identified and meta-analysis was performed according to doses. The study was registered with PROSPERO (CRD42023392454).

**Results:**

Six hundred and twelve patients from four randomized controlled trials were enrolled. The results demonstrated that FFA at 0.2, 0.4, or 0.7 mg/kg/d showed significantly greater efficacy compared to placebo in terms of at least 50% reduction (*p* < 0.001, *p* < 0.001, *p* < 0.001) and at least 75% reduction (*p* < 0.001, *p* = 0.007, *p* < 0.001) in monthly seizure frequency from baseline. Moreover, significantly more patients receiving FFA than placebo were rated as much improved or very much improved in CGI-I by both caregivers/parents and investigators (*p* < 0.001). The most common treatment-emergent adverse events were decreased appetite, diarrhea, fatigue, and weight loss, with no valvular heart disease or pulmonary hypertension observed in any participant. For dose comparison, 0.7 mg/kg/d group presented higher efficacy on at least 75% reduction in seizure (*p* = 0.006) but not on at least 50% reduction. Weight loss (*p* = 0.002), decreased appetite (*p* = 0.04), and all-cause withdrawal (*p* = 0.036) were more common in 0.7 mg/kg/d group than 0.2 mg/kg/d. There was no statistical difference in other safety parameters between these two groups.

**Conclusion:**

The higher range of the licensed dose achieves the optimal balance between efficacy, safety, and tolerability in patients with DS and LGS.

**Clinical trial registration:**

https://www.crd.york.ac.uk/PROSPERO/, identifier CRD42023392454.

## 1 Introduction

Despite the existence of multiple anti-epileptic drug regimens, drug-resistant epilepsy (DRE) remains a major problem (1, 2). Dravet syndrome (DS) and Lennox–Gastaut syndrome (LGS) are common drug-resistant developmental and epileptic encephalopathies in infancy and early childhood; both have diverse seizure types and are often accompanied by serious cognitive deterioration and psychiatric and intellectual impairment (3–5). Severe decline in quality of life tends to leave patients with DS or LGS desperate for novel antiseizure medications (ASMs) to improve their condition. Currently, the first-line medication options for DS include valproate (VPA) and clobazam (CLB) (5), whereas VPA, lamotrigine, and topiramate (TPM) are usually the preferred choices for LGS (4, 6, 7). Considering the limited therapeutic effect of these ASMs, some relevant clinical trials are ongoing, which have led to the preliminary verification of newly discovered antiseizure drugs, such as stiripentol (STP) and cannabidiol (CBD) for DS, and rufinamide, CBD, and felbamate for LGS, followed by their approval in several countries and regions (8–12). More recently, fenfluramine (FFA) has gained prominence as a possible alternative to treat both DS and LGS, as well as other DREs (13–20).

FFA, a serotonergic medication, is an amphetamine derivative and a racemic mixture of D- and L-enantiomers (6). Initially, a high dose of FFA was widely accepted in combination with phentermine (Fen-Phen) for the treatment of obesity in 1984 and gained significant popularity in overweight women (21, 22). However, owing to accumulating evidence that its chronic use could result in the increasing incidence of valvular heart disease (VHD) and pulmonary hypertension (PAH), it was withdrawn from the market in 1997 (23–27). At the same time, as Aicardi et al. (28) and Clemens et al. (29) presented early data from single case reports and small case series demonstrating a significant reduction in seizure frequency when FFA was added to the existing treatment regimen, the anti-epileptic effects of FFA have gradually received increasing attention. Subsequently, clinical trials of low-dose FFA to treat DRE, including DS, LGS, CDKL5 deficiency disorder (CDD), and sunflower syndrome were gradually underway (9, 14, 30–33). In this systematic review and meta-analysis, we analyzed the results of large double-blind placebo-controlled trials, which preliminarily confirmed the efficacy and safety of low-dose FFA in DREs at doses up to 0.7 mg/kg/day (maximum: 26 mg/day) to provide further evidence for the optimal use of FFA in DREs (16–19).

## 2 Materials and methods

This meta-analysis was performed following the Preferred Reporting Items for Systematic Reviews and Meta-Analyses (PRISMA) (11). The study was registered with PROSPERO (CRD42023392454).

### 2.1 Data sources and search strategy

All randomized, placebo-controlled, double-blind trials were identified by searching PubMed, Embase (via Ovid), and Web of Science before December 1st, 2023, with no language restrictions. The search terms included (1) fenfluramine, fintepla, pondimin and (2) drug-resistant epilepsy, refractory epilepsy, Lennox–Gastaut syndrome, Dravet syndrome, severe myoclonic epilepsy in infancy, west syndrome, infantile spasm, CDKL5 deficiency disorder, Doose syndrome, Rasmussen Syndrome, Sturge–Weber syndrome. The two groups of keywords were combined with Boolean “AND” and synonymous terms were combined with Boolean “OR.” The reference lists of the full-text reports were screened to identify other relevant studies. Any disagreements were resolved by consensus among the reviewers.

### 2.2 Inclusion criteria

#### 2.2.1 Study design

Randomized controlled trial (RCT).

#### 2.2.2 Subjects

Patients diagnosed with DRE, including DS and LGS, without VHD or PAH before enrollment.

#### 2.2.3 Intervention

Different doses of FFA (0.2, 0.4, or 0.7 mg/kg/day) were administered, with a placebo as the control group.

#### 2.2.4 Outcomes

Detailed data on responder events, withdrawal events, and treatment-related adverse events (TEAEs) are available.

### 2.3 Exclusion criteria

#### 2.3.1 Study design

Non-RCTs, including retrospective and observational studies, case reports, and open-label studies were excluded.

#### 2.3.2 Subjects

Non-DRE.

#### 2.3.3 Outcome

No detailed data were accessible to assess efficacy and safety.

### 2.4 Data extraction and quality assessment

Two reviewers (Y.C.X and D.C) independently extracted the following data from the eligible studies: first author, year of publication, NCT registration number, trial region, patient characteristics (age range, sex, and ASMs), study duration, and necessary outcome events (responder, withdrawal, and TEAE). The evidence was evaluated according to the guidelines for assessing the risk of bias in the Cochrane Handbook (34). Any disagreements were resolved by consensus among the reviewers.

### 2.5 Data synthesis and analysis

The primary outcome was a reduction in monthly seizure frequency (MSF; convulsive seizure in DS and drop seizure in LGS) of at least 50% from baseline, while the secondary outcomes were a reduction in MSF of at least 75% from baseline, near seizure freedom (seizure frequency ≤ 1), seizure freedom, caregiver/parent or investigator- rated Clinical Global Impression Improvement (CGI-I) scales. TEAEs were selected as safety endpoints. The analysis was performed by calculating the risk ratios (RR), and 95% confidence intervals (95% CI) of the data (34). The results were visualized using forest plots. Statistical heterogeneity was estimated using the *I^2^* statistic as follows: *p*>0.10 was considered as low heterogeneity, and fixed effects model was used; if *p* ≤ 0.10, a fixed- or random-effects model was adopted for *I*^2^ < 40% or ≥ 40%, respectively (35). Subgroup analysis was conducted to investigate the differences in efficacy and adverse effects of the three doses of FFA in DS. Sensitivity analyses were conducted through leave-one-out meta-analyses to assess the influence of individual studies on the overall treatment effect estimate. A *p*-value <0.05 was considered significant for all analyses, and all tests were two-tailed. All statistical analyses were performed using Stata 15.1 software.

## 3 Results

### 3.1 Literature search

**Figure 1:**
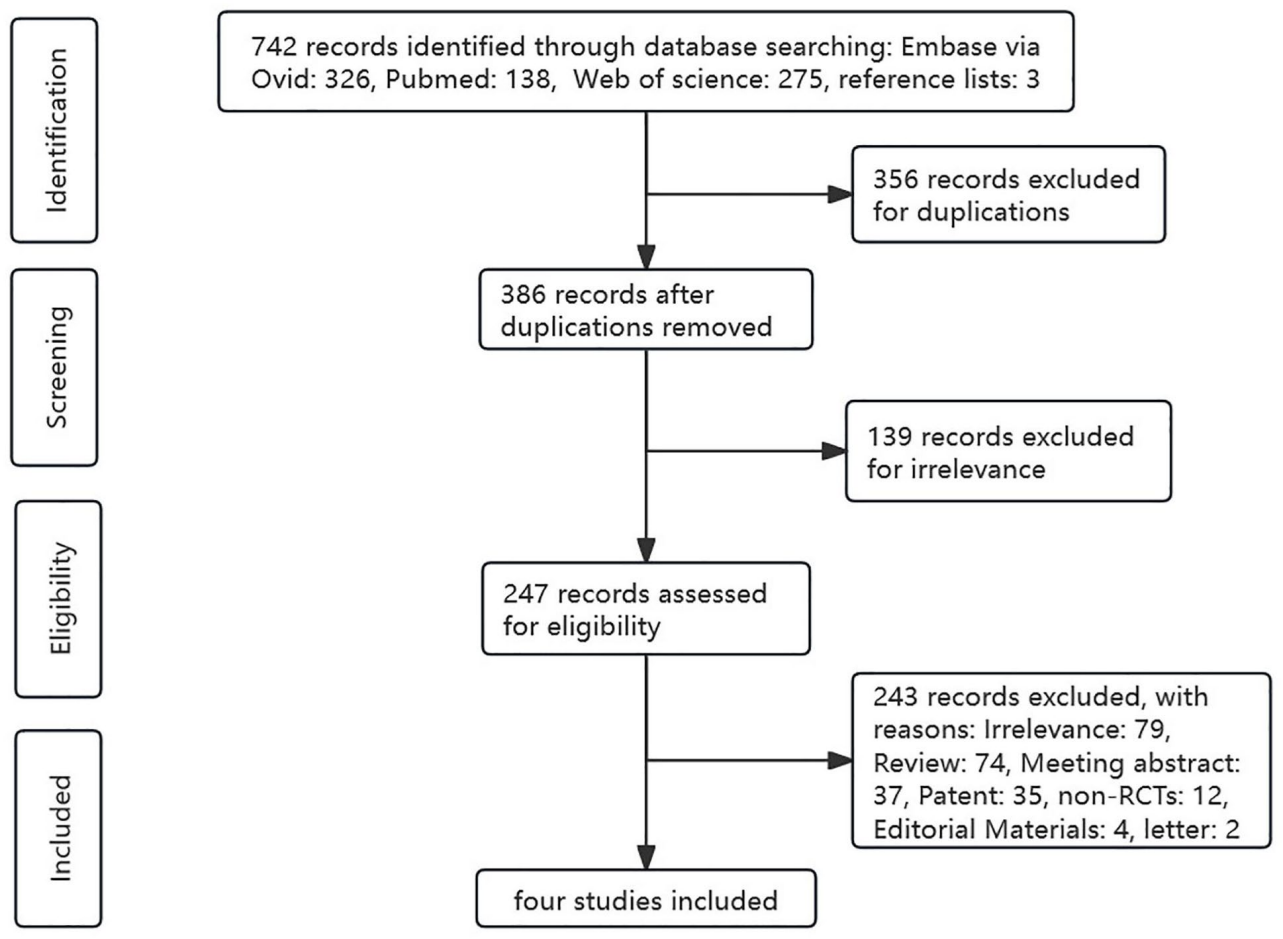
The study selection process for this meta-analysis.

We initially obtained 742 results from a literature search, of which 326 from Embase (via Ovid), 138 from PubMed, 275 from Web of Science, and 3 from reference lists, among which 356 were excluded as they were duplicates. After reviewing the titles, abstracts, and keywords, 139 articles were excluded because the content was not directly related. Then, 243 trials were excluded by irrelevance, or non-target study type or outcomes. Finally, four RCTs were included in this meta-analysis. The detailed literature screening process is shown in Figure 1.

### 3.2 Study characteristics

A total of 612 participants were included in this study. The four retrieved trials were all phase 3 multicenter RCTs with sample sizes of 263, 119, 87, and 143, respectively. The four studies reported the efficacy and safety of three doses [0.2 mg/kg/d (17–19), 0.4 mg/kg/d (16), and 0.7 mg/kg/d (17–19)] of FFA as adjunctive therapy in DS and LGS. In four RCTs, Nabbout et al. (16) included patients with DS receiving STP, while Lagae et al. (18) specifically excluded patients with inadequate pharmacokinetic data of FFA-STP drug interactions. The most common ASMs recorded by the participants were VPA, CLB, TPM, and levetiracetam. The entire treatment period was 14 or 15 weeks and consisted of two phases: a titration period (2 or 3 weeks) and a maintenance period (12 weeks). The baseline characteristics of the included studies are presented in Table 1.

**Table 1:** The baseline characteristics of the included studies.

Study	Study design	Epilepsy type	Intervention	Sex (M / F) Age (mean ± SD)	Course of treatment	Concomitant ASMs	Adverse events
Knupp et al. (15)	RCT Phase 3	LGS	FFA 0.7 mg/kg/d FFA 0.2 mg/kg/d PBO	46/41, 14 ± 8 46/43, 13 ± 8 54/33, 13 ± 7	Titration 2w Maintenance 12w	Valproate (all forms), Clobazam, Lamotrigine, Levetiracetam, Rufinamide	Decreased appetite, Somnolence, Fatigue, Pyrexia, Diarrhea, Vomiting
Lagae et al. (16)	RCT Phase 3	DS	FFA 0.7 mg/kg/d FFA 0.2 mg/kg/d PBO	21/19, 8.8 ± 4.4 22/17, 9.0 ± 4.5 21/19, 9.2 ± 5.1	Titration 2w Maintenance 12w	Valproate (all forms), Clobazam, Lamotrigine, Levetiracetam	Decreased appetite, Diarrhea, Nasopharyngitis, Lethargy, Somnolence, Pyrexia, Fatigue, Seizure, Vomiting, Weight decrease, Fall
Nabbout et al. (18)	RCT Phase 3	DS	FFA 0.4 mg/kg/d PBO	23/20, 8.8 ± 4.6 27/17, 9.4 ± 5.1	Titration 3w Maintenance 12w	Stiripentol, Clobazam Valproate, Topiramate, Levetiracetam	Decreased appetite, Pyrexia, Fatigue, Diarrhea, Nasopharyngitis, Lethargy, Bronchitis
Sullivan et al. (17)	RCT Phase 3	DS	FFA 0.7 mg/kg/d FFA 0.2 mg/kg/d PBO	22/26, 9.4 ± 5.3 24/22, 9.6 ± 4.4 27/21, 9.0 ± 4.3	Titration 2w Maintenance 12w	Clobazam, Levetiracetam, Topiramate, Valproate (all forms)	Diarrhea, Pyrexia, Fatigue, Nasopharyngitis, Blood glucose decreased, Decreased appetite, Somnolence, Tremor

CI, confidence interval; FFA, Fenfluramine; MSF, monthly seizure frequency; PBO, Placebo; RR, risk ratio.

### 3.3 Quality assessment of the included studies

According to the Cochrane Risk of Bias Assessment Tool, random sequence generation, allocation concealment, blinding of participants and personnel, blinding of outcome assessment, outcome data integrity, reporting bias, and other biases were fully considered and assessed. In the studies of Lagae et al. (18), Knupp et al. (19), and Sullivan et al. (17), we considered “other bias” as “unclear risk.” As Lagae et al. (18) mentioned in their study, the presence of side effects known to be associated with FFA might lead patients or caregivers suspicious of receiving FFA, thus inducing subjective feelings and an inability to accurately report the seizure frequency. Nevertheless, Nabbout et al. (16) noted no relevant evidence regarding the above conjecture according to the results of post-hoc analysis. All of the remaining items were considered to be of low risk and high quality.

### 3.4 Primary efficacy outcomes

**Figure 2:**
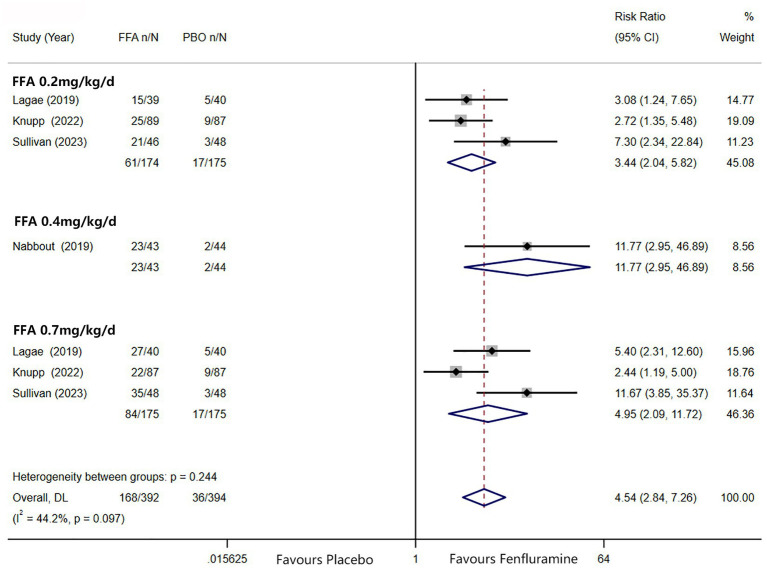
Forest plot of at least 50% reduction in MSF from baseline. CI, confidence interval; FFA, fenfluramine; MSF, monthly seizure frequency; PBO, placebo.

A total of 168 patients in the FFA group (42.9%) and 19 patients in the placebo group (8.7%) showed a reduction in MSF of at least 50% from baseline. Different doses of FFA [0.2 mg/kg/d: RR =3.44, 95%CI: 2.04, 5.82], *p* < 0.001; [0.4 mg/kg/d: RR = 11.77, 95%CI: 2.95, 46.89], *p* < 0.001; [0.7 mg/kg/d: RR = 4.95, 95%CI: 2.09, 11.72], *p* < 0.001 all presented better efficacy over placebo, while the pooled RR of 4.54 (95% CI: 2.84, 7.26) indicated a significant antiseizure effect of FFA over placebo (*p* < 0.001) (Figure 2).

### 3.5 Secondary efficacy outcomes

**Figure 3:**
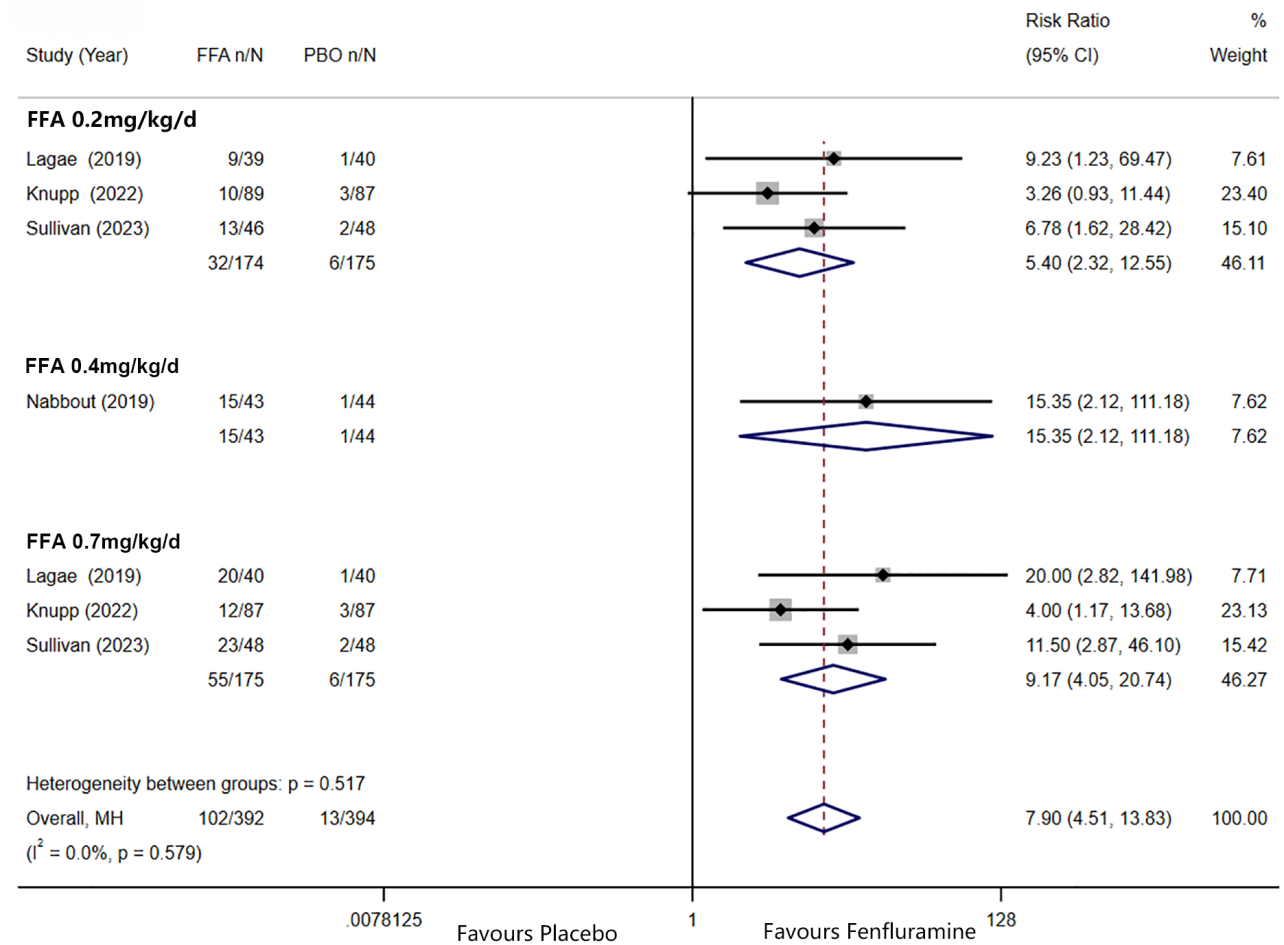
Forest plot of at least 75% reduction in MSF from baseline. CI, confidence interval; MSF, monthly seizure frequency; FFA, fenfluramine; PBO, placebo.

**Figure 4:**
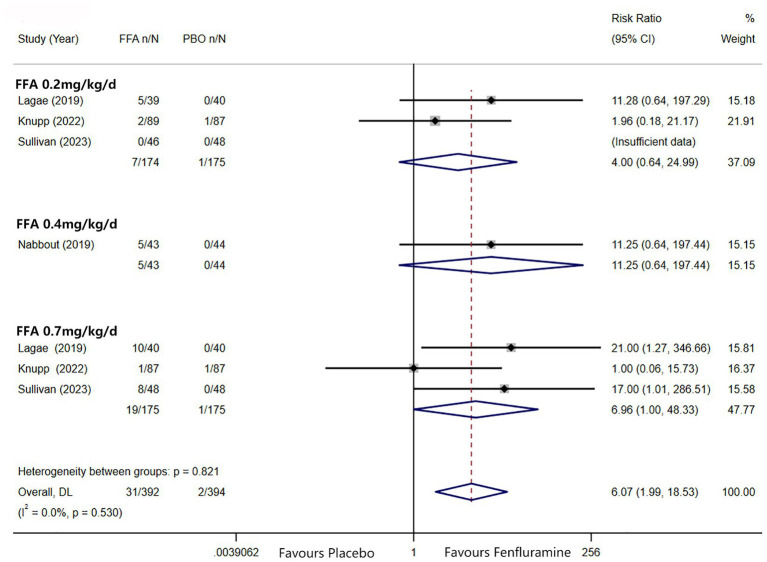
Forest plot of near seizure freedom. CI, confidence interval; FFA, fenfluramine; MSF, monthly seizure frequency; PBO, placebo.

**Figure 5:**
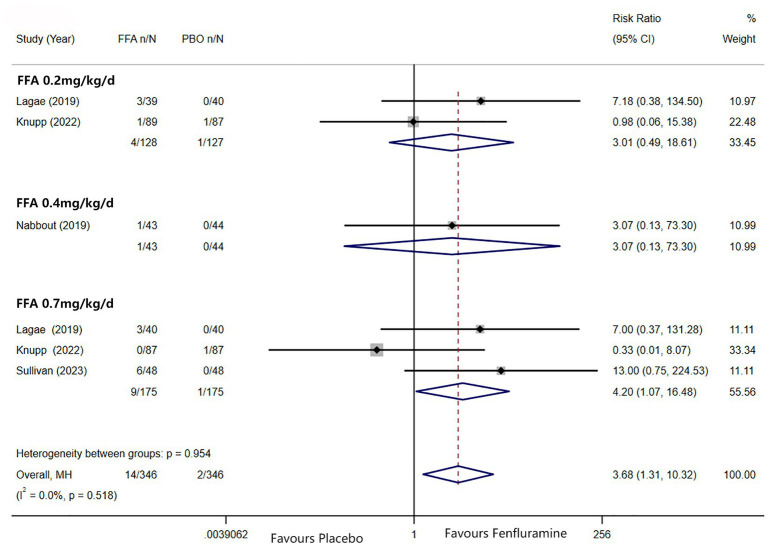
Forest plot of seizure freedom. CI, confidence interval; FFA, fenfluramine; MSF, monthly seizure frequency; PBO, placebo.

A reduction in MSF of at least 75% from baseline was achieved in 102 patients (26.0%) in the FFA group versus seven patients (3.2%) in the placebo group. Different doses of FFA [0.2 mg/kg/d: RR = 5.40, 95%CI: 2.32, 12.55], *p*<0.001; [0.4 mg/kg/d: RR = 15.35, 95%CI: 2.12, 111.18], *p* = 0.007; 0.7 mg/kg/d: RR = 9.17, 95%CI: 4.05, 20.74], *p*<0.001 all showed a certain number of advantages compared to the placebo. The overall RR was 7.90 [(95%CI: 4.51, 13.83), *p* < 0.001] and the corresponding forest plots are shown in Figure 3. We observed no significant differences between different doses of FFA and placebo as to near seizure freedom (seizure frequency ≤ 1) (*p* = 0.137, *p* = 0.098, *p* = 0.050) but the overall efficacy showed a significant difference [RR = 6.07, (95%CI: 1.99, 18.53)] (Figure 4). Similarly, no significant differences between different doses of FFA and placebo were observed in seizure freedom (*p* = 0.373, *p* = 0.489, *p* = 0.261) but the overall efficacy showed a significant difference [RR = 3.68, (95%CI: 1.31, 10.32)] (Figure 5).

Regarding the CGI-I scale, more patients in the FFA group (0.2, 0.4, 0.7 mg/kg/d) were considered by the caregivers/parents (*p* < 0.001, *p* = 0.208, *p* < 0.001) and investigators (*p* < 0.001, *p* = 0.008, *p* < 0.001) to have a much improved or very much improved rating. The total RR were [4.52, (95% CI: 3.18, 6.41), *p* < 0.001] and [4.44, (95% CI: 3.14, 6.29), *p* < 0.001], respectively.

### 3.6 Safety

Table 2 shows the adverse events of different doses of FFA recorded in the four studies. TEAEs occurred in 350 patients (89.3%) in the FFA group and 173 patients (79.0%) in the placebo group [RR (95% CI) = 1.14 (0.99, 1.31), *p* = 0.08], although with no statistically significant difference. Among the adverse effects, the two groups showed significant differences in decreased appetite [RR (95% CI) = 3.34 (2.14, 5.20), *p* < 0.001], diarrhea [RR (95% CI) = 2.60 (1.48, 4.53), *p* = 0.0007], fatigue [RR (95% CI) = 2.27 (1.04, 4.96), *p* = 0.04] and weight loss (loss ≥7%) [RR (95% CI) = 4.60 (1.90, 11.14), *p* = 0.0007]. Nevertheless, no statistically significant difference was found between the FFA group and placebo group in dropping out for any reason [RR (95% CI) = 1.24 (0.56, 2.74), *p* = 0.60] and side effects or lack of efficacy [RR (95% CI) = 1.80 (0.69, 4.67), *p* = 0.23]. In the group of 0.2 mg/kg/d FFA, significant differences were observed in decreased appetite [RR (95% CI) = 2.43 (1.38, 4.28), *p* = 0.003] and diarrhea [RR (95% CI) = 2.61 (1.34, 5.09), *p* = 0.005]. In the group of 0.4 mg/kg/d FFA, no significant differences were observed in adverse effects. And In the group of 0.7 mg/kg/d FFA, significant differences were observed in TEAE [RR (95% CI) = 1.23 (1.05, 1.44), *p* = 0.01], decreased appetite [RR (95% CI) = 4.01 (2.38, 6.78), *p*<0.001], diarrhea [RR (95% CI) = 2.25 (1.14, 4.44), *p* = 0.02], and weight loss [RR (95% CI) = 5.76 (1.86, 17.82), *p* = 0.002], but no significant difference were found between the FFA group and placebo group in dropping out for any reason and side effects or lack of efficacy (All *p*>0.05).

**Table 2:** Adverse events.

Adverse effect	Number of studies	FFA	PBO	RR [95%CI]	*p value*
*FFA 0.2 mg/kg/d*					
TEAE	3	175	175	1.16 [0.97,1.38]	0.10
SAE	2	135	135	1.58 [0.41, 6.07]	0.51
Blood glucose decreased	1	46	48	1.91 [0.77,4.75]	0.16
Decreased Appetite	3	175	175	2.43 [1.38, 4.28]	**0.002**
Diarrhea	3	175	175	2.61 [1.34, 5.09]	**0.005**
Pyrexia	3	175	175	0.96 [0.55, 1.67]	0.88
Fatigue	3	175	175	1.45 [0.54, 3.92]	0.46
Weight loss	3	175	175	2.78 [0.70, 11.07]	0.15
*FFA 0.4 mg/kg/d*					
TEAE	1	43	44	1.02 [0.95, 1.11]	0.57
SAE	1	43	44	0.88 [0.32, 2.40]	0.8
Blood glucose decreased	1	43	44	3.07 [0.66, 14.38]	0.15
Decreased appetite	1	43	44	3.89 [1.60, 9.48]	**0.003**
Diarrhea	1	43	44	3.41 [1.01, 11.55]	0.05
Pyrexia	1	43	44	2.81 [0.97, 8.16]	0.06
Fatigue	1	43	44	2.81 [0.97, 8.16]	0.06
*FFA 0.7 mg/kg/d*					
TEAE	3	175	175	1.23 [1.05, 1.44]	**0.01**
SAE	2	135	135	2.59 [0.95, 7.06]	0.06
Blood glucose decreased	1	46	48	1.33 [0.5, 3.55]	0.57
Decreased Appetite	3	175	175	4.01 [2.38, 6.78]	**<0.0001**
Diarrhea	3	175	175	2.25 [1.14, 4.44]	**0.02**
Pyrexia	3	175	175	0.79 [0.26, 2.44]	0.68
Fatigue	3	175	175	2.15 [1.09, 4.23]	**0.03**
Weight loss	3	175	175	5.76 [1.86, 17.82]	**0.002**

CI, confidence interval; FFA, Fenfluramine; PBO, Placebo; RR, risk ratio. Bold: the corresponding *p*-value was < 0.05 and the difference was statistically significant.

### 3.7 Comparison of different doses

We also separately conducted a comparative analysis of 0.2 mg/kg/d and 0.7 mg/kg/d FFA about their efficacy and safety (only Knupp et al.’s study had 0.4 mg/kg/d group and was not appropriate for dose analysis considering its unique on add-on with STP). In this comparison, 0.7 mg/kg/d showed significantly higher efficacy than 0.2 mg/kg/d in ≥75% reduction in MSF [RR (95% CI) =1.65 (1.16, 2.44), *p* = 0.006], whereas no statistical difference was found in terms of ≥50% reduction in MSF [RR (95% CI) =1.39 (0.95, 2.02), *p* = 0.086] with random-effects model (*I^2^* = 56.2%, *p* = 0.102). However, we found the heterogeneity was mainly derived from Knupp et al.’s study (19) and the total effect of ≥50% reduction in MSF became significant as we removed it from the pooled effect [RR (95% CI) = 1.66 (1.25, 2.19)]. For other efficacy outcomes, there was no statistical difference comparing 0.7 mg/kg/d with 0.2 mg/kg/d in near seizure freedom and complete seizure freedom (All *p*>0.5).

Regarding the CGI-I scale, more patients in the 0.7 mg/kg/d group were considered by the caregivers/parents [RR (95% CI) =1.44 (1.10, 1.88), *p* = 0.008] and investigators [RR (95% CI) =1.53 (1.16, 2.02), *p* = 0.002] to have a much improved or very much improved rating.

Decreased appetite, diarrhea, fatigue, and weight loss were commonly reported adverse events in all four studies. In comparison, 0.7 mg/kg/d FFA caused more patients to get weight loss [RR (95% CI) =1.53 (1.16, 2.02), *p* = 0.002] and decreased appetite [RR (95% CI) = 2.05 (1.03, 4.50), *p* = 0.04] than 0.2 mg/kg/d FFA. No statistically significant difference was observed in the comparison of occurrence of all TEAE, diarrhea, and fatigue (All *p*>0.5). On withdrawal rate, 0.7 mg/kg/d FFA had a higher risk for dropping out for any reason than 0.2 mg/kg/d [RR (95% CI) =2.30 (1.05, 5.03), *p* = 0.036] but no significant difference was found in dropping out for side effects or lack of efficacy between the two different doses [RR (95% CI) =2.60 (0.94, 7.20), *p* = 0.066].

### 3.8 Subgroup analysis and sensitivity analysis

**Figure 6:**
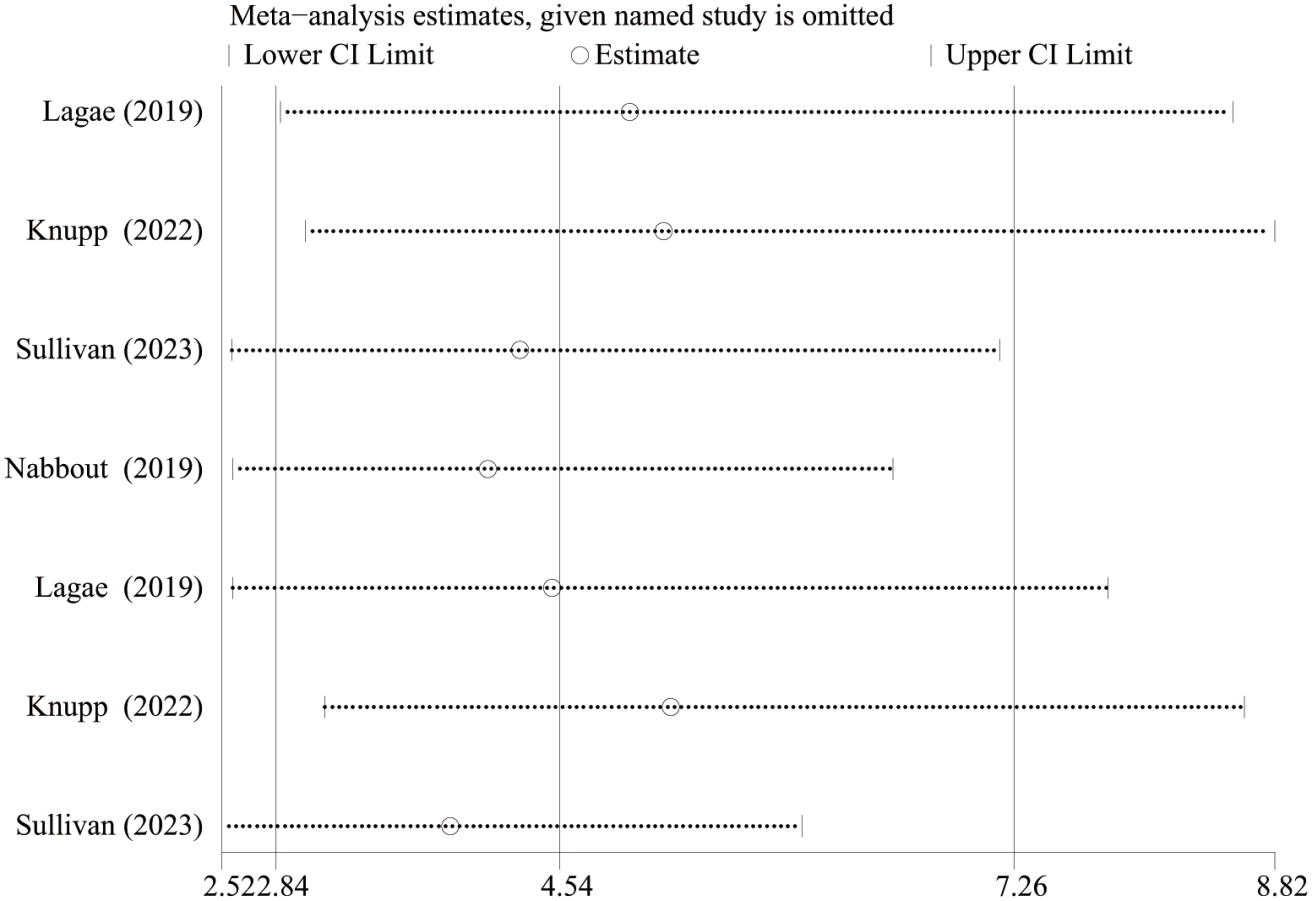
Sensitivity analysis of at least 50% reduction in MSF from baseline. CI, confidence interval.

As Lagae et al. (18), Nabbout et al. (16) and Sullivan et al. (17) all explored the efficacy and safety of FFA in DS, we excluded one article about FFA in the treatment of LGS and further analyzed the difference in efficacy (≥ 50% and ≥ 75% reduction in MSF, near seizure freedom, and complete seizure freedom) and safety (drop out for any reason and side effects or lack of efficacy) between different doses of FFA for DS. And statistically significant differences were found in all efficacy outcomes. The overall pooled RR [95%CI] were 6.83[4.32, 10.81], 11.62[5.49, 24.60], 14.66[3.55, 60.60], 6.96[1.58, 30.63] for ≥50% and ≥ 75% reduction in MSF, near seizure freedom, and complete seizure freedom, respectively. However, no significant differences were found in TEAE, SAE, dropping out for any reason and dropping out for side effects or lack of efficacy between different doses of FFA in DS (All *p*>0.5). We performed sensitivity analyses for at least ≥50% reduction in MSF which showed high heterogeneity between studies (*p*<0.10), and the total effect was within the range of 95% CI (2.84–7.26), which suggested the results were stable and reliable in this meta-analysis (Figure 6).

## 4 Discussion

FFA is a novel adjunctive ASM for DRE (36). Serotoninergic activity, which raises serotonin (5-hydroxytryptamine) levels in the brain by inducing their release from vesicular storage sites and also by inhibiting their reuptake to increase GABAergic signaling, is thought to be the primary anti-epileptic mechanism of FFA (37–39). In addition, FFA also reacts with sigma-1 receptors which may bind to N-methyl-D-aspartate (NMDA) and G-protein coupled receptors to control Ca^2+^ influx to decrease glutamatergic excitability and then exerts a potential anti-epileptic effect (22, 39, 40). In this study, we investigated the efficacy, safety, and tolerability of different doses of add-on FFA in the treatment of both DS and LGS.

Consistent with the findings reported by Damavandi et al. (41), our meta-analysis observed a significant reduction in seizure frequency among patients treated with fenfluramine, reinforcing the drug’s efficacy in managing DS and LGS. Furthermore, our dose-specific analysis echoes the discussions by Dini et al. (42) on the critical need for tailored treatment approaches, particularly in optimizing dosing regimens to balance efficacy with safety. When concentrated on different doses of FFA, 0.7 mg/kg/d FFA was more effective than 0.2 mg/kg/d FFA in reducing at least 75% seizure frequency. No statistical difference was found in terms of ≥50% reduction in MSF and heterogeneity was mainly derived from Knupp et al. (19) and the total effect changed when left it out (RR [95% CI] = 1.66[1.25, 2.19]). As the 0.4 mg/kg/d dose was used in only one study by Nabbout et al. (16) and the included patients were treated with STP simultaneously, we cannot draw a hasty conclusion on its efficacy over other doses. We also took the CGI-I scores of patients rated by investigators and caregivers/parents into consideration. More investigators and parents believed that patients in the 0.7 mg/kg/d FFA group experienced better conditions, which further reflected the convincing anti-epileptic efficacy of FFA.

Significant difference was found in the TEAE between FFA and the placebo group. In comparison, FFA was more likely to cause decreased appetite, diarrhea, fatigue, and weight loss, which may be associated with the earliest clinical use of FFA as an appetite suppressant (43). A higher dose of FFA was associated with a higher risk for side effects such as decreased appetite, and weight loss. Nevertheless, the overall incidence of withdrawal events due to adverse effects or lack of efficacy between the 0.7 and 0.2 mg/kg/d FFA was not statistically significant. Therefore, considering the safety and tolerability of FFA, a higher range of licensed doses may be a better choice to treat DRE (DS and LGS).

In four trials, none of the participants developed VHD or PAH. Moreover, a subsequent 3-year open-label trial on DS treated with low-dose FFA (maximum dose: 0.7 mg/kg/d) conducted by Agarwal et al. (44) found that none of the 327 patients progressed to VHD or PAH at any time within the trial phase, suggesting a good long term safety profile of the adjunctive FFA for DS and LGS. Meanwhile, drug–drug interaction-related studies showed no distinct impact of FFA on the pharmacokinetics of anti-epileptic drugs that are commonly used for DS and LGS. However, as STP may affect the metabolism of FFA (45–47), Nabbout et al. (16) reduced the daily dose of adjunctive FFA when combined with STP. The optimal dose of FFA with STP still requires further investigation.

Apart from DS and LGS, studies on FFA for the treatment of other DREs are also ongoing (15). A small sample size study of FFA for CDD conducted by Devinsky et al. (14) recruited six patients with CDD and initially demonstrated that 0.4 mg/kg/d and 0.7 mg/kg/d FFA decreased seizure frequency with no distinct adverse effects. An open-label trial preliminarily validated the therapeutic effect of low-dose FFA (no more than 0.7 mg/kg/d) in nine patients with sunflower syndrome, with ≥70% reduction in seizure frequency achieved in six of the nine patients who completed a 3-month core study (33). No serious adverse effects, such as lethal cardiac disease, were observed in any of these studies. Meanwhile, another study applying FFA as an adjunct to the treatment of five different types of developmental and epileptic encephalopathies (DEEs) (SYNGAP1 Encephalopathy, STXBP1 Encephalopathy With Epilepsy, Inv Dup (15) Encephalopathy, Multifocal or Bilateral Malformations of Cortical Development, Continuous Spike and Waves During Slow Sleep) is also enrolling patients in a non-controlled clinical trial (NCT05232630), which focuses on assessing seizure frequency, intensity, and duration before and after FFA treatment. Moreover, “non-epileptic outcomes,” such as variations in cognitive activity, level of alertness, impulsivity/self-control, gait stability, and other alterations may also be detected during the interview and physical examination. These studies are expected to demonstrate the crucial role of low-dose FFA in DRE and promote its clinical application.

Our study has some limitations. As the number of RCTs included was small, there was a lack of comprehensive persuasiveness. Efficacy of FFA administered at 0.4 mg/kg/day was explored in only one study (16). Evidence for the efficacy, safety, and tolerability of FFA comes from short-term treatments. Thus, long-term outcomes are essential to further explore the optimal dose of FFA. In addition, few studies evaluated the impact of FFA on cognitive functions with DRE. Preliminary results show that FFA was associated with improvement in everyday executive functions in 28% of children with DS (48). As current studies on FFA do not focus much on the “non-epileptic outcomes” of epileptic encephalopathies, such as cognitive, psychiatric, and intellectual outcomes, it may be a vital direction for future analysis.

## 5 Conclusion

Taken together, the higher range of the licensed dose prescribed for seizure control showed significantly higher efficacy and fair safety for DS and LGS. Decreased appetite and weight loss seem dose-dependent. No VHD or PAH were observed even at the highest dose.

## Data availability statement

The original contributions presented in the study are included in the article/supplementary material, further inquiries can be directed to the corresponding author.

## Author contributions

YX: Conceptualization, Formal analysis, Investigation, Methodology, Writing – original draft. DC: Conceptualization, Formal analysis, Investigation, Methodology, Writing – review & editing. LL: Conceptualization, Supervision, Writing – review & editing.
